# Tempol effect on epithelial-mesenchymal transition induced by hyperglycemia

**DOI:** 10.15171/jnp.2017.01

**Published:** 2016-08-03

**Authors:** Mohammad Jafari, Farahnaz Dadras, Hamid Reza Ghadimipour, Mohamad Ali Seif Rabiei, Farhad Khoshjou

**Affiliations:** ^1^Pathology ward, Hamadan University of Medical Sciences, Hamadan, Iran; ^2^Nephrology Ward, Department of Internal Medicine, Iran University of medical Sciences, Tehran, Iran; ^3^Department of Community Medicine, Hamdan University of Medical Sciences, Hamadan, Iran; ^4^Urology and Nephrology Research Center, Hamadan University of Medical Sciences, Hamadan, Iran

**Keywords:** Tempol, Epithelial mesenchymal transition, Diabetic nephropathy

## Abstract

**Background:**

One of common mechanisms in pathophysiology of chronic kidney diseases is epithelial-mesenchymal transition (EMT). On the other hand oxidative stress has been known to participate in kidney damage of diabetic nephropathy (DN).

**Objectives:**

We studied if tempol, a well-known antioxidant agent, can ameliorate EMT in DN induced in male rats.

**Materials and Methods:**

Twenty-seven male rats were equally divided in to 4 groups. Group I (control or C), group II (diabetic or D), group III (T) rats which was given tempol (100 mg/kg/day) by gavages for 28 days and group IV (D&T) was diabetic rats that also received same dose of tempol. After treatment, their kidneys were studied by immunohistochemicalstaining.

**Results:**

Pathological changes in the kidney were detected concurrently with increasing kidney weight and urinary albumin excretion. In addition, EMT indices, i.e. decline of E-cadherin and increase of α SMA staining were significantly emerged in the renal tubular cells of diabetic group and were partially modified in diabetic group which were simultaneously treated by tempol.

**Conclusions:**

Tempol can modify, but not significantly, EMT induced by DN.

**Implication for health policy/practice/research/medical education:**

Diabetic nephropathy (DN) is the major etiology of chronic kidney disease. On the other hand, oxidative stress has been well known to be involved in its pathophysiology. Besides, epithelial- mesenchymal transition (EMT) is a common finding in kidney injury. In current study, we tried to figure out if administration of tempol, an antioxidant agent, can modify EMT in DN.

## 1. Background

Epithelial cells are polarized with their basal exterior connected to basement membrane and their apical side facing the lumen of a tubular structure. Sideways, they interact with neighboring epithelial cells, forming a pavement of cells (1).

Epithelial-mesenchymal transition (EMT) includes loss of intercellular junctions between epithelial cells, their detachment from a basement membrane, loss of epithelial phenotype markers and gaining of mesenchymal markers and cell movement (2).

Tian et al showed that the shape and cytoskeleton of proximal tubular epithelial cells undergo notable restructuring in response to TGF-β (3). They lose their cobblestone morphology and become spindle shaped. This is accompanied by downregulation of expression of E-cadherin, a key constituent of adherens junctions, de novo expression of α-SMA and reformation of the actin microfibrillar which is characteristic of myofibroblasts.

### 1.1. EMT in human kidney diseases

In more than 100 biopsies from different human renal diseases, Rastaldi et al showed that, there was regular evidence for acquisition of mesenchymal markers in tubular cells (4).

### 1.2. EMT in diabetic nephropathy

Diabetic nephropathy (DN) is the leading cause of end-stage renal disease in the world. Glomerulosclerosis and interstitial fibrosis are the typical features of DN (5). It has been shown that EMT formation in tubular epithelial cells plays a key role in the progression for tubulointerstitial fibrosis in DN (6).

Myofibroblasts play foremost role in progression of renal fibrosis in diabetic kidney disease. Cells expressing α-smooth muscle actin (α-SMA), the accepted marker of myofibroblasts, are placed primarily in the renal interstitium (7).

On the other hand, the number of myofibroblasts is inversely correlated with renal function in DN (8). However, the origin of myofibroblasts remains uncertain. One widespread hypothesis is that myofibroblasts are derived from epithelial myofibroblast transition.

Evidence for EMT is emerging in animal models of DN (9). In human biopsy samples of DN, EMT is a common feature, as well (10). Podocyte also shows EMT phenomena in DN (11).

Role of oxidative stress in pathophysiology of DN has been well established. Tempol, on the other hand, is a well-known antioxidative agent. It is a superoxide (SOD) mimetic and has been broadly studied in animal models of DN (12,13). In present study, we tried to figure out, where tempol administration can modifies EMT, induced by DN in rats.

## 2. Objectives

Bearing in mind that, oxidative stress has major impact on pathophysiology of DN, we were going to figure out if tempol, a well-known antioxidant agent, can ameliorate EMT in DN induced in male rats.

## 3. Materials and Methods

### 3.1. Animals and treatments

Adult male Wistar rats weighing 180–250 g maintained on a 12-hour light/dark cycle with free access to tap water and standard laboratory chow were used. Animals were randomly divided into 4 groups of 7 animals and treated for 4 weeks by gavages. This experimental model of rats made diabetic with streptozotocin (STZ) injection has been validated in previous studies (14). Diabetes disease was induced by only a single intraperitoneal (i.p.) injection of STZ (60 mg/kg body weight) which was prepared by citrate buffer, pH 4.5. The fasting blood glucose (FBS) levels were determined three days after STZ injection by using a strip-operated blood glucose sensor. Animals were considered diabetic if plasma glucose levels exceeded 250 mg/dL. The groups were as follows: control group, tempol group (100 mg/kg/day), diabetic group, and diabetic and also tempol group. At the end of the treatment, 24 hours after the last dose of treatment, animals were killed, urine and blood samples were collected in tubes and serum was isolated quickly and kept frozen at -80°C.

### 3.2. Experimental protocols

#### 3.2.1. Kidney parameters

Blood urea nitrogen (BUN), creatinine (Cr) levels and urine albumin and Cr were estimated using an automated biochemistry machine ‎according to the standard procedure of kits.

#### 3.2.2. Histopathological analysis

The kidneys were fixed in paraformaldehyde and embedded in paraffin. Transverse sections (4 µm) were cut, and hematoxylin-eosin (H&E) staining then were performed.

#### 3.2.3. Immunohistochemistry

Briefly, 3-µm sections were placed into histosol to remove the paraffin, rehydrated through graded ethanol, and incubated for 30 minutes with normal rat serum. The sections were then incubated with anti-E-cadherin (1: 50, Santa Cruz), anti-α -smooth muscle actin (α-SMA, 1: 100, Santa Cruz) overnight (18 hours) at 4°C. The following day, the sections were incubated with a biotinylated swine anti-rabbit IgG antibody (1: 200, DAKO, Denmark), followed by treatment with an avidin-biotin peroxidase complex (1: 200). Localization of the peroxidase conjugates was achieved by using diaminobenzidine tetrahydrochloride as a chromogen. The sections were counterstained in Mayer’s hematoxylin. The sections incubated in normal rabbit serum (1: 10) served as the negative controls.

#### 3.2.4. Pathologic assessment

All of the specimens were evaluated by two pathologist separately. Staining severity in every section of the specimen were scored in at least 7 randomly selected non-overlapping fields at ×40 magnifications.

They were scored based on this grading system from: 1+to 4+. Grade 1+ represents very weak staining, grade 2+, weak staining; grade 3+, moderate staining and grade 4+ means severe staining.

### 3.3. Ethical issues

The research followed the tenets of the Declaration of Helsinki. The research was approved by ethical committee of Hamadan University of Medical Sciences. Prior to the experiment, the protocols were confirmed to be in accordance with the Guidelines of Animal Ethics Committee of Hamadan University of Medical Sciences.

### 3.4. Statistical analysis

Values were expressed as mean ± standard error (SE). The statistically significant differences between groups were analyzed by one-way analysis of variance (ANOVA) with Bonferroni multiple-comparison post hoc test.

## 4. Results

**Figure 1:**
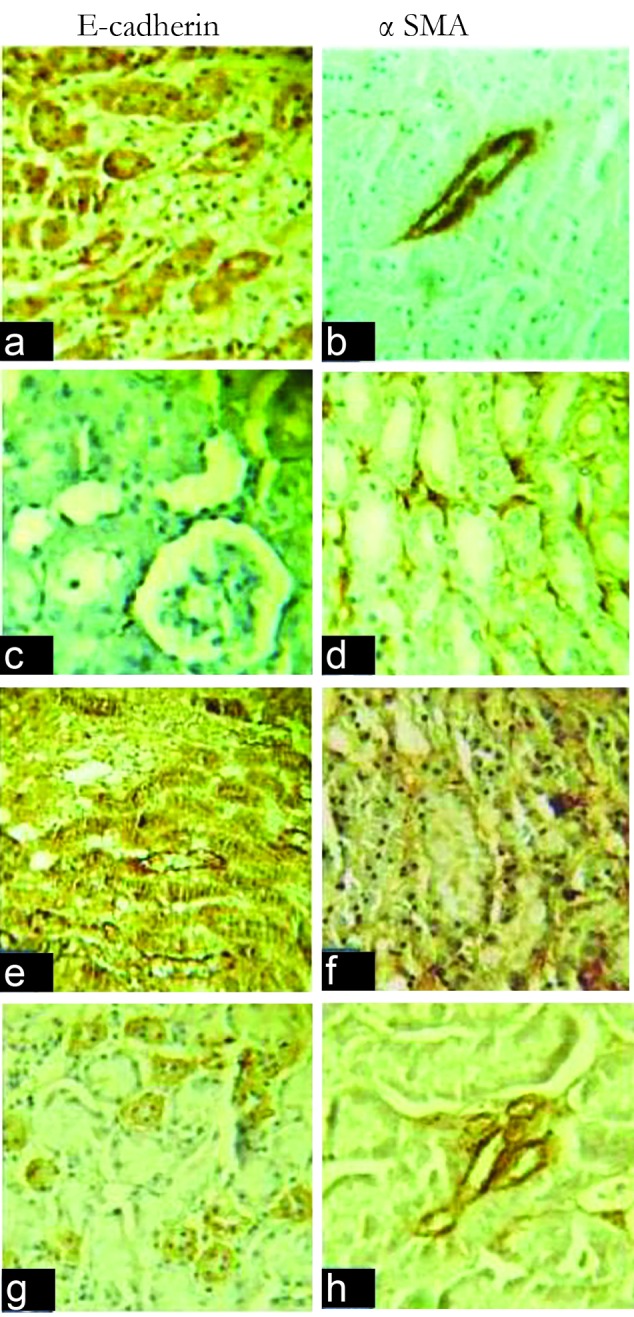
First row (a &b): control group, second row (c &d) diabetic group, third row (e & f) diabetic and tempol group, fourth row (g & h) tempol group.

After 3 days of follow up, diabetes was induced in rats which were injected STZ intraperitoneally. DN also confirmed by significant rising of albumin/creatinine ratio (ACR) in diabetic (group D) rats after 28 days follow up (#8 folds). Serum urea, but not serum creatinine, increased in group D significantly. Comparing four groups for staining severity using α-SMA or E-cadherin showed a statistically significant difference between four groups with both methods (Table 1, Figure 1).

**Table 1:** Mean staining score in four groups with** α-**SMA and E-cadherin

**Group**	**Mean score (SD)**	**P**
α-SMA staining		
1 (Control)	1.16 (0.4)	
2 (Diabetic)	3.37 (0.51)	<0.001*
3 (Diabetic & tempol)	2.66 (0.51)	NS
4 (Tempol)	1.33 (0.51)	NS
E-cadherin staining		
1 (Control)	2.83 (0.75)	
2 (Diabetic)	1.37 (0.51)	0.017*
3 (Diabetic & tempol)	2.00 (0.89)	NS
4 (Tempol)	2.00 (0.89)	NS

*Comparing to group 1(control), NS; not significant.

Pathologic assessment of immunohistochemical staining sections for E-cadherin using Bonferroni as a post hoc method showed that there is significant difference between control group and diabetic ones (*P*=0.011). There is also significant difference between above-mentioned groups in α-SMA stained sections (*P*<0.001). However, there was not significant difference between diabetic (group D) and diabetic group which treated by tempol (group D&T). It includes both E-cadherin and α-SMA stained sections.

## 5. Discussion

Pathophysiologic studies of DN have been showed that oxidative stress has major rule in (15). On the other hand, EMT has been known as a common finding in kidney injury, including DN (16). Therefore, a couple of studies have been allocated to figure out if antioxidant agents, can modify kidney injury in diabetic milieu (17).

In current study, a group of diabetic rats treated by tempol and its effects on EMT induced by hyperglycemia assessed by two markers E-cadherin and α-SMA.

Significant difference between control and diabetic groups, showed by these markers, indicated that, EMT outbreaks in early period of DN (28 days after induction of diabetes in rats). Nevertheless, tempol administration, despite its effects on oxidative parameters, cannot modify EMT significantly. To the best of our knowledge, this is the first time that tempol effect on EMT has been studied.

## 6. Conclusions

EMT is an early finding in DN. Tempol, a well-known antioxidant cannot modifies EMT significantly even when administered at the beginning of diabetes.

## Limitations of the study

We did not measure and compare glomerular filtration rate (GFR) in different groups of rats.

## Conflicts of interest

The authors declare that they have no conflicting interest.

## Authors’ contribution

MJ and MRG reviewed pathology slides. FD collected the data, MASR achieved statics and FK was the study supervisor, contributed to all aspect of the study and provided the final manuscript. All authors read and approved the paper.

## Funding/Support

This paper extracted of the thesis titled by “Tempol effects on DN in male rats”. This research project was approved and supported by research Deputy of Hamadan University of medical sciences and the number of this project is 9211013647.
